# Frequency of Macroprolactinemia in Hyperprolactinemic Women Presenting with Menstrual Irregularities, Galactorrhea, and/or Infertility: Etiology and Clinical Manifestations

**DOI:** 10.1155/2013/478282

**Published:** 2013-09-30

**Authors:** Alfredo Leaños-Miranda, Karla Leticia Ramírez-Valenzuela, Inova Campos-Galicia, Rosario Chang-Verdugo, Lizbeth Zarela Chinolla-Arellano

**Affiliations:** ^1^Research Unit in Reproductive Medicine, Unidad Médica de Alta Especialidad, Hospital de Ginecología y Obstetricia, Luis Castelazo Ayala, Instituto Mexicano del Seguro Social, Don Luis No. 111, Col. Nativitas, 03500 México, DF, Mexico; ^2^Clinical Laboratory, Unidad Médica de Alta Especialidad, Hospital de Ginecología y Obstetricia, Luis Castelazo Ayala, Instituto Mexicano del Seguro Social, México, DF, Mexico

## Abstract

*Aim*. To determine the frequency of macroprolactinemia, its etiology, and the clinical manifestations in patients with hyperprolactinemia presenting with menstrual irregularities, galactorrhea, and/or infertility who were attended by the gynecology-endocrinology service. *Methods*. In a cross-sectional study, 326 hyperprolactinemic women were tested for serum prolactin (PRL) concentrations before and after chromatographic separation (gel filtration and affinity with protein G) and extraction of free PRL with polyethylene glycol (PEG). *Results*. Sera from 57 patients (17.5%) were found to have macroprolactinemia. The presence of macroprolactinemia was attributable to anti-PRL autoantibodies in 54 (94.7%) patients. The median serum PRL levels were similar in patients with or without macroprolactinemia (42.0 versus 38.1 ng/mL). In contrast, patients with macroprolactinemia had lower serum-free PRL levels (median 9.2 versus 31.7 ng/mL, *P* < 0.001). Patients without macroprolactinemia had a higher frequency of galactorrhea and abnormal pituitary imagine findings (*P* < 0.002). *Conclusions*. We can conclude that macroprolactinemia should be considered as a benign variant, and it must be ruled out in women presenting with menstrual irregularities, galactorrhea, and/or infertility in order to investigate other causes different than hyperprolactinemia. Serum PRL precipitated with PEG is a convenient and simple procedure to screen for the presence of macroprolactinemia.

## 1. Introduction

Prolactin (PRL) is a polypeptide hormone primarily secreted by the anterior pituitary gland.

The presence of several PRL isoforms in serum and other biological fluids has been clearly established. The major circulating isoform of PRL is a 23 kDa single polypeptide chain (monomeric PRL), which comprises up to 80% of the total PRL in serum from normal subjects and the majority of patients with hyperprolactinemia (HPRL). In addition, there are two other PRL isoforms that display higher molecular weights, referred to as big PRL (45–50 kDa) and big big PRL (>100 kDa) and also known as macroprolactin [1]. The presence of these isoforms has been attributed to formation of aggregates of monomeric PRL with different glycosylation degrees and binding of PRL to serum protein in circulation, mainly to anti-PRL autoantibody of IgG isotype [2–5]. These structural modifications may distinctly affect the biological and immunological properties of the hormone [5]. Immunometric methods, which are commonly used to determine serum PRL, are largely blind to changes in the patterns and proportions of PRL isoforms, which may influence both the net *in vivo* biological activity of the hormone and the clinical features of PRL-related disease states. To date, it is well recognized that the molecular heterogeneity of PRL is present in sera from hyperprolactinemic subjects. Predominant presence of big big PRL, a phenomenon termed macroprolactinemia (MPRL), has been reported in 15 to 46% of subjects with HPRL [6–13]. Although the nature of MPRL is still unclear, much evidence indicates that big big PRL is mostly an IgG-23 kDa PRL complex (i.e., anti-PRL autoantibody-monomeric PRL) [3, 14, 15]. Macroprolactin is big enough to be confined to vascular spaces, and therefore HPRL develops due to slower serum clearance of macroprolactin [16] and also due to lack of negative feedback, because macroprolactin cannot freely access the hypothalamus [14]. In addition, macroprolactin displays low biological activity *in vitro* [5, 17]. Independently of the nature of big big PRL (i.e., due or not to anti-PRL autoantibodies), clinical symptoms of HPRL, such as amenorrhea and galactorrhea in women and impotence in men, are usually less frequent or even absent in patients with MPRL [2, 4, 18]. However, several patients with MPRL cannot be distinguished from true hyperprolactinemic patients on the basis of clinical features alone [11, 12, 19].

Since signs and symptoms of HPRL are nonspecific and relatively common, it is possible that some patients with MPRL experience these signs and symptoms coincidentally, but unrelated to PRL, resulting in misdiagnosis and inappropriate treatment [20].

Since there is scarce information on the frequency of MPRL in hyperprolactinemic women with signs and symptoms related to hyperprolactinemia, the aim of the present work was to study MPRL frequency and its etiology in hyperprolactinemic women presenting with menstrual irregularities, galactorrhea, infertility, or/and alterations in libido who were attended by the gynecology and endocrinology service of a third level care hospital, as well as to determine the utility of the percentage of serum PRL precipitated with polyethylene glycol (PEG) assay in the detection of MPRL and to establish the ideal cut-off point for this test.

## 2. Material and Methods

The study protocol was approved by the Human Ethical Committee and Medical Research Council of the Instituto Mexicano del Seguro Social. Written informed consents were obtained from all study participants.

All participant women were patients attended by the Gyneco-Endocrinology outpatient clinic of our hospital; those with serum PRL levels greater than 25 ng/mL were included in the study. A clinical history was obtained with special reference to the presence of menstrual irregularities, galactorrhea, infertility, or alterations in libido. Between six and twelve months later, from clinical chart, we obtained information on imaging investigations, diagnoses, and treatments used.

A venous blood sample was drawn between 07:00 and 08:00 a.m., under basal conditions and without hormonal or drug stimulus. Sera were stored at −70°C until being used.

### 2.1. Determination of Direct or Total Serum PRL Levels

PRL concentration in serum was measured by an ultrasensitive enzyme immunoassay previously described [21]. This particular method has shown a high reliability for detecting serum PRL independently of its isoform composition; in this assay the presence of anti-PRL autoantibodies does not interfere with the test results, yielding concentration values of 5–25 ng/mL in normal conditions. The sensitivity of the assay was 0.018 ng/mL, and the withinassay and between assay coefficients of variation were 5.7% and 6.8%, respectively. 

### 2.2. Determination of Serum Free or Monomeric PRL Levels

Free PRL was extracted from the serum using PEG, as previously described [22].

### 2.3. Gel Filtration Chromatography and Affinity Chromatography

Gel filtration was performed on Sephacryl 200 HR column (60 × 1 cm) (GE Biosciences), using a previously described procedure [22]; recovery of PRL was 110 ± 8.4%, on average. Affinity chromatography for IgG was performed using 1 mL protein-G Sepharose columns (Gammabind G, GE-Healthcare, Chalfont St. Giles, UK), as described [14, 22]. Immunoreactive PRL present in eluate fractions was determined by the same ultrasensitive enzyme immunoassay. Three patterns were observed in gel filtration chromatography, determined by analysis of the area under the PRL elution curve: (1) exclusive or predominant pattern of MPRL (≥50% of big big PRL), (2) exclusive or predominant pattern of monomeric PRL (≥50% of little PRL), and (3) variable pattern, without a predominant pattern of the previously mentioned possibilities.

In addition, serum samples were considered to contain anti-PRL autoantibodies when the retained percentage of PRL by the protein-G Sepharose column was above 4.2% this value represents the mean + 3 SD obtained from sera of 40 healthy pregnant women without MPRL and who had little PRL as the predominant circulating species of PRL (≥95% as confirmed by size exclusion chromatography).

### 2.4. Statistical Analysis

The significance of differences between continuous variables was determined by the nonpaired Student's *t*-test (or Mann-Whitney *U* test for nonnormally distributed variables). Differences between categorical variables were determined by the Chi-square test with Yates's continuity correction (or Fisher's exact test for small samples). The lineal relationship between serum total PRL levels and the percentage of bound PRL-IgG (anti-PRL autoantibody), as well as between relative amounts of big big PRL determined by gel filtration chromatography and the percentage of PRL precipitated with PEG was assessed by the Pearson correlation coefficient. A receiver operating characteristic curve for the percentage of serum PRL precipitated with PEG assay was generated. The area under the curve was calculated to establish the ideal cut-off point for this test for the detection of MPRL. A 2-tailed *P* < 0.05 was considered statistically significant.

## 3. Results

### 3.1. General Description

The study sample consisted of 326 hyperprolactinemic patients. Mean age was 31.4 ± 2.9 years (range 20–42). Indications for testing serum PRL levels include the following: menstrual irregularities, galactorrhea, infertility, and/or alterations in libido (Table 1). Median serum PRL was 38.3 ng/mL (range 25.5–1,860.0 ng/mL). Among of these patients with HPRL, in 87 (26.7%) there was an identifiable condition that could account for the increased PRL levels (secondary HPRL): 61 patients were attributable to overt or subclinical hypothyroidism, evaluated on the basis of normalization of TSH and PRL concentrations with L-thyroxine, except in six patients who had MPRL and remained hyperprolactinemic despite the normalization of TSH levels; 22 patients were attributable to prolactinomas, and 4 patients were attributable to the use of medications. In the remaining 239 patients (73.3%), no cause could be identified that would explain the presence of HPRL (idiopathic HPRL).

### 3.2. Frequency of MPRL and Its Etiology

**Figure 1:**
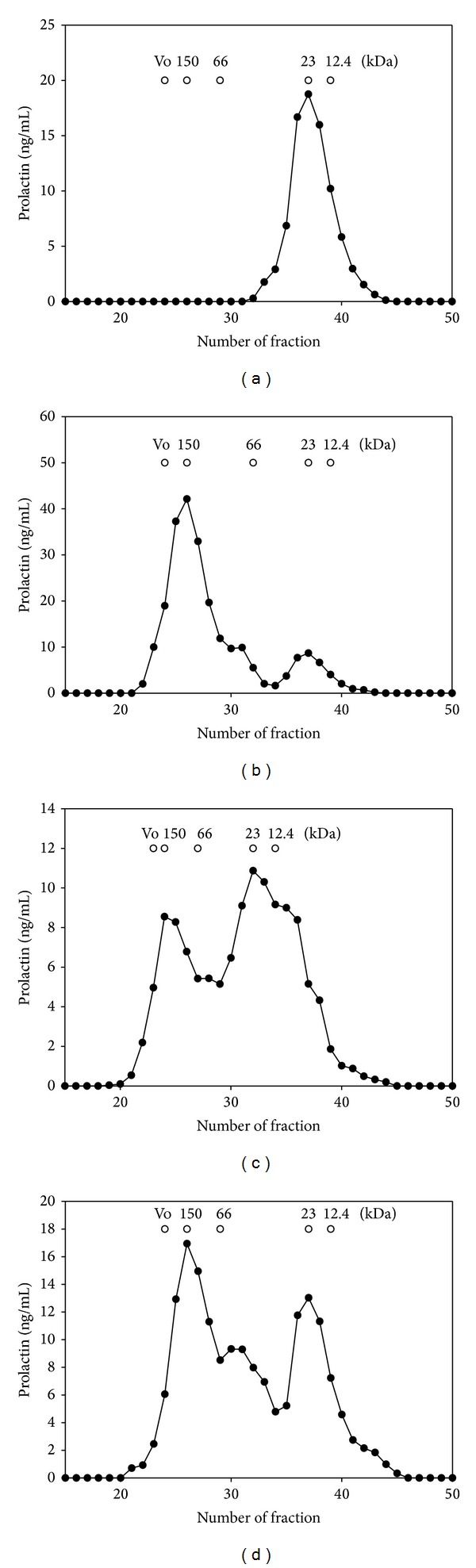
Representative gel filtration profiles of immunoreactive PRL in sera from hyperprolactinemic patients on a Sephacryl HR 200 column (60 × 1 cm). Samples (1 mL) were applied on the column, and the fractions of 900 *μ*L were collected. (a) Exclusive or predominant pattern of monomeric PRL (little PRL); (b) exclusive or predominant pattern of macroprolactinemia (big big PRL); ((c) and (d)) variable patterns.

According to gel filtration profiles of immunoreactive PRL, sera from 57 hyperprolactinemic patients were found to have an exclusive or predominant pattern of MPRL (17.5%; confidence interval (CI), 95%, 13.4–21.6%) (Table 1, Figure 1). The frequency of MPRL among patients with secondary HPRL (all with hypothyroidism) was 6.9% (95% CI 1.6–12.2%). In contrast, 51 of the 239 patients with idiopathic HPRL had MPRL (21.3%, 95% CI 16.1–26.5%; *P* = 0.014).

Sera from two patients displayed a variable pattern (0.6%) with a percentage of MPRL of 40.8 and 44.6%, respectively, and the remaining 267 patients (81.9%) had an exclusive or predominant pattern of monomeric PRL (23 kDa, Figure 1).

**Figure 2:**
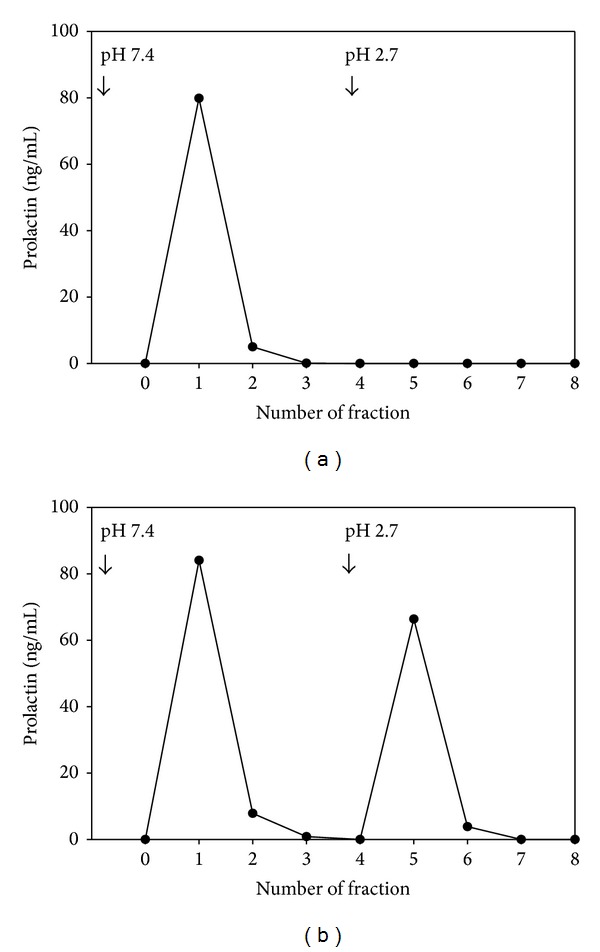
Representative affinity chromatography profiles of immunoreactive PRL in sera from one patient without anti-PRL autoantibody (a) and one patient whose test was positive for anti-PRL autoantibody (b) on a protein-G sepharose column (1 mL). Samples (1 mL) were applied on the column, and fractions of 1 mL were collected.

In 54 of 57 patients with MPRL, a significant amount of immunoreactive PRL was retained on the protein G-sepharose column (42.5 ± 3.9% versus 0.8 ± 1.4% (*P* < 0.001) in sera without MPRL) (Figure 2). In the 3 remaining sera with MPRL, almost all immunoreactive PRL passed through the protein G-sepharose column, and it was similar in sera without MPRL. Then, the frequency of anti-PRL autoantibodies was 94.7% (95% CI 88.9–100%). Data also showed a positive correlation (*r* = 0.63, *P* < 0.001) between the percentage of serum PRL retained on protein G-sepharose column and serum PRL levels. 

### 3.3. Characterization of Patients with MPRL

To characterize hyperprolactinemic patients with and without MPRL, a comparison was made among their clinical and laboratory variables (Table 1). We found that the median of total PRL levels in patients with MPRL was not different from that of patients without MPRL (42.0 ng/mL (range 26.0–268.6) versus 38.1 ng/mL (range 25.5–1,860.0), *P* = 0.27); in contrast, the median of free PRL levels was significantly lower (9.2 ng/mL (range 2.2–22.2) versus 31.7 ng/mL (range 25.5–1,840.0) *P* < 0.001). Gel filtration chromatography profiles of immunoreactive PRL in sera from patients with MPRL showed that much of (72.0 ± 10.0%) PRL was eluted as big big PRL (approximately 150 kDa). By contrast, in patients without MPRL, the 23 kDa form of PRL remained the predominant species identified (96.0 ± 8.7%). Likewise, when serum IgG-bound PRL was extracted by affinity chromatography, a significant amount (38.5 ± 4.5%) of immunoreactive PRL was coeluted with IgG fractions in samples from patients with MPRL, while marginal amounts of PRL were detected in IgG fractions from patients without MPRL (0.8 ± 1.4%, *P* < 0.001).

On the other hand, the frequency of galactorrhea was significant higher in patients without MPRL than in those with MPRL (*P* = 0.002), whereas there was no difference in frequency of oligomenorrhea, amenorrhea, infertility, or alterations in libido between the two groups. Finally, six of the 57 patients with MPRL had secondary causes of HPRL (10.5%); in contrast, 81 of the 269 patients without MPRL had secondary causes of HPRL (30.1%, *P* = 0.004). 

### 3.4. Follow-Up of Patients

Imaging investigations (computed tomography or magnetic resonance imaging) were performed in 16/57 (28.1%) patients who had MPRL and in 36/269 (13.4%) patients without MPRL. The frequency of abnormalities was exclusively found in patients without MPRL (11 were normal, 18 revealed a microadenoma, and 7 revealed a macroadenoma). All imaging studies were normal in patients with MPRL.

Forty-four (77.2%) patients with MPRL and 147 (54.6%) patients without MPRL were treated with dopamine agonists (*P* = 0.003). Symptomatic improvement occurred in 79 of 147 (53.7%) patients without MPRL and in 4 of 44 (9.1%) patients with MPRL (*P* < 0.001). A diagnosis of polycystic ovary syndrome was made in 22 of 269 (8.2%) patients without MPRL and in 4 of 57 (7.0%) patients with MPRL (*P* = 0.93). 

### 3.5. Relationship between the Percentage of Serum PRL Precipitated by PEG and the Percentage of MPRL by Gel Filtration Chromatography

**Figure 3:**
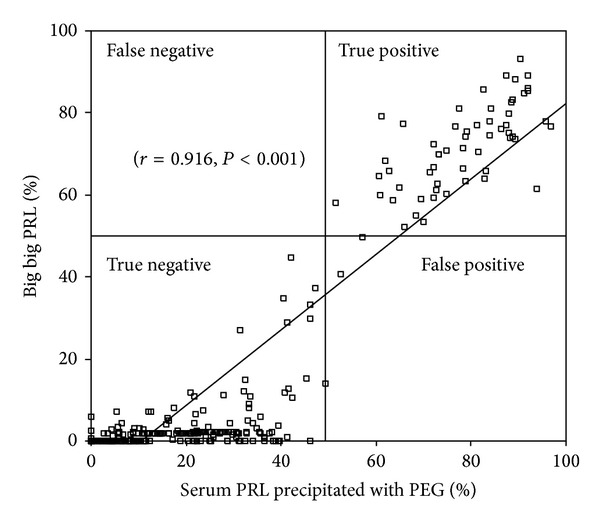
Relationship between the percentages of big big PRL (as determined by gel filtration chromatography) present in serum samples from 326 hyperprolactinemic patients (269 without macroprolactinemia and 57 with macroprolactinemia) and percentages of serum PRL precipitated by PEG. The horizontal line denotes the 50% limit for percentage of macroprolactinemia, and the vertical line is set to 49.2% of serum PRL precipitated by PEG (see Section 3).

We observed a significant positive correlation between the percentage of serum PRL precipitated by PEG (PRL in serum-PRL in supernatant after PEG precipitation/PRL in serum ×100) and the percentage of MPRL by gel filtration chromatography (as gold standard to detect the presence of MPRL, *r* = 0.92, *P* < 0.001) (Figure 3). The area under the receiver operating characteristic curve was 0.998 (95% CI 0.988–1, *P* < 0.001). The optimal cut-off point was ≥49.2%; this cut-off yielded a sensitivity of 100% (95% CI 93.7–100), specificity of 99.3% (95% CI 97.3–99.9), and positive and negative predictive values of 95.8% (95% CI 87.5–98.7) and 99.8% (95% CI 98.2–100), respectively. At this cut-off point, positive and negative likelihood ratios were 107.1 (95% CI 31.2–367.8) and 0.009 (95% CI 0.001–0.14), respectively. As shown in Figure 1, there were two false-positive samples; however, these serum samples displayed a variable pattern by gel filtration chromatography with a percentage of MPRL of 40.8 and 44.6%, respectively. 

## 4. Discussion

Several publications have associated asymptomatic HPRL with the predominance of MPRL [7, 18, 19, 23]. The frequency reported of MPRL in hyperprolactinemic subjects using similar methodologies has been 15–46% [6–13]. However, the frequency of MPRL in hyperprolactinemic women with signs and symptoms related to elevated serum PRL levels is unknown. This study demonstrates that 17.5% of our selected hyperprolactinemic women studied (i.e., presenting with menstrual irregularities, galactorrhea, infertility, or alterations in libido) had significant MPRL. This finding is in accordance with a recently published study, which showed a frequency that is similar (11.5%, 95% CI 6.9–16.1%) in a group of selected hyperprolactinemic patients with infertility [24]. Although the nature of MPRL is still unclear in some subjects with HPRL, the present study clearly indicates that the etiology of MPRL in the majority of patients is due to anti-PRL autoantibodies, mainly in those with very high serum PRL levels without a proven cause of the HPRL [4, 25, 26]. Other minor causes of MPRL have been attributed to formation of aggregates of monomeric PRL with different glycosylation degrees [4].

It has been suggested that the anti-PRL autoantibody itself is the cause of HPRL; to explain this, the following observations have been proposed as follows: (1) the PRL-antibody complex is eliminated more slowly from the bloodstream than free PRL [16, 27]; (2) the PRL-IgG complex could block feedback mechanisms in the endocrine system, resulting in a low level of serum PRL to the hypothalamus and pituitary; this point may also be relevant in the presentation of active PRL to cells bearing PRL receptors [22]; and (3) there exist a positive correlation between the proportion of PRL bound to IgG and serum PRL levels ([3, 22, 27], as in the present study).

Measurements of serum immunoreactive PRL concentrations in hyperprolactinemic subjects do not always correlate with the clinical findings. In fact, several authors have reported that asymptomatic HPRL is frequently associated with the presence of molecular heterogeneity, particularly the predominant presence of MPRL [8–10, 18, 28]. However, other authors have reported that patients with MPRL cannot be distinguished from patients with HPRL, but without MPRL on the basis of clinical features alone [12, 13]. These discrepancies may be due to population studied (as our patients included), the use of different immunoassays, or criteria for definition of MPRL. In this study, although the frequency of galactorrhea was significantly higher in patients without MPRL than in patients with MPRL, these differences are not sufficient to distinguish between these two entities. Moreover, since signs and symptoms of HPRL are nonspecific and relatively common, it is possible that some patients with MPRL experience these signs and symptoms coincidentally, but unrelated to PRL, resulting in misdiagnosis and inappropriate investigation and treatment as reported in this study and by prior studies [7, 10, 11]. Indeed, there are reports of patients who underwent pituitary surgical exploration [29, 30]. Moreover, the findings that serum free PRL (monomeric PRL) levels from patients with MPRL were ≤22.2 ng/mL and that all images of studies were normal, as well as the fact that the symptomatic improvement after the treatment with dopamine agonists occurred in only 9.1% of patients suggest that signs and symptoms experienced by our patients studied were coincidental rather than being attributable to a true HPRL.

Abnormal findings on pituitary imaging were more frequent in patients without MPRL than in patients with MPRL (69.4 and 0%, resp.), and these results are consistent with those of other studies [8, 10, 13]. On the other hand, the finding that the treatment with dopamine agonists was prescribed in 77.2% of patients with MPRL is consistent with previous reports ranging from 76.5 to 86.7% [7, 10].

As being consistent with previous studies [31, 32], the results of this study indicate that precipitation of MPRL by PEG is an effective technique for detecting MPRL. The percentage of MPRL with values of ≥49.2% precipitated with PEG (PRL recovery of ≤50.7%) reflected an ideal cut-off point for detecting exclusive predominant pattern of MPRL by gel filtration chromatography (as gold standard) with high accuracy. Furthermore, values below 49.2% of precipitation of serum PRL can be interpreted as virtually excluding MPRL and solely the presence of the monomeric PRL form circulating in the blood. The least common cases with precipitation between 49.2% and lower than 60.0% require gel filtration chromatography to characterize the predominant molecular form of PRL. In this vein, another definition of MPRL has been proposed requiring that concentrations of free PRL fall in the range of serum samples from normoprolactinemic healthy subjects treated with PEG [10, 11]. This approach is reasonable because it can identify patients who need further investigations and treatments for HPRL.

In accordance to previous studies [10, 11, 13, 33], the present results provide more support to the notion that MPRL can be a benign variant and that treatment with dopamine agonists, imaging investigations, or prolonged follow-up is not necessary. However, the patients with MPRL and menstrual irregularities, galactorrhea, infertility, or alteration in libido should be investigated for other causes different than HPRL. 

In summary, we have demonstrated that macroprolactinemia is a prevalent cause of HPRL among women presenting with menstrual irregularities, galactorrhea, infertility, or alterations in libido attended by gynecology and endocrinology clinic, and we confirmed that the main etiology of MPRL is due to presence of anti-PRL autoantibodies. Macroprolactinemia should be taken into account as a probable cause of HPRL in order to avoid misdiagnosis and unnecessary investigations and treatment. The percentage of serum PRL precipitated by PEG provides a good estimation of the predominant presence of big big PRL as determined by gel filtration chromatography. Nevertheless, the diagnostic performance of the percentage of serum PRL precipitated by PEG should be validated locally by each laboratory because of the variability in laboratory methods used to measure PRL and the criteria to establish cut-off points. 

## Figures and Tables

**Table 1:** Demographic and clinical data, serum total and free immunoreactive prolactin (PRL) levels, distribution of PRL immunoreactivity in three fractions obtained after gel filtration, and percentage of retained PRL in affinity chromatography in hyperprolactinemic patients according to the absence or presence of macroprolactinemia (MPRL).

Variable	Without MPRL (n = 269)	With MPRL (n = 57)	*P* value
Age, yrs, mean ± SD	33.6 ± 8.9	32.9 ± 9.1	0.62^b^
Body mass index, mean ± SD	27.4 ± 4.8	27.7 ± 4.1	0.72^b^
Direct PRL, ng/mL, median (range)	38.1 (25.5–1,860.0)	42.0 (26.0–268.6)	0.27^c^
Free PRL, ng/mL, median (range)	31.7 (25.5–1,840)	9.2 (2.2–22.2)	<0.001^c^
Big big PRL (%), mean ± SD	2.9 ± 1.2	72.0 ± 11.2	<0.001^b^
Big PRL (%), mean ± SD	2.5 ± 2.4	3.1 ± 2.1	0.85^b^
Little PRL (%), mean ± SD	96.0 ± 8.7	24.9 ± 10.1	<0.001^b^
IgG-bound PRL (%), mean ± SD^a^	0.8 ± 1.4	38.5 ± 4.5	<0.001^b^
Anti-PRL autoantibodies (%)	0 (0)	54 (94.7)	<0.001^d^
Oligomenorrhea or amenorrhea (%)	116 (44.8)	28 (49.1)	0.50^d^
Galactorrhea (%)	91 (33.8)	7 (12.3)	0.002^d^
Infertility (%)	85 (31.6)	21 (36.8)	0.54^d^
Alterations in libido (%)	13 (4.8)	1 (1.8)	0.50^e ^
Secondary hyperprolactinemia (%)	81 (30.1)	6 (10.5%)	0.004^d^
Polycystic ovarian syndrome (%)	22 (8.2)	4 (7.0)	0.93^e^
Dopamine agonist prescribed (%)	147 (54.6)	44 (77.2)	0.003^d^

^a^PRL retained in protein G-Sepharose column (PRL retained/PRL nonretained + PRL retained × 100%).

^b^Nonpaired Student's *t*-test, ^c^Mann-Whitney *U*-test, ^d^ *χ* ^2^ test, and ^e^Fisher's exact test.
